# Supplementary material for: Mapping the evolution of fertility support policies in China: A content and instrumental analysis
Source: PLoS One. 2025 Oct 9;20(10):e0332137. doi: 10.1371/journal.pone.0332137 (PMC12510515; doi:10.1371/journal.pone.0332137)
Supplement: S1 Appendix — (ZIP) [file pone.0332137.s001.zip › S1 Appendix. 226 original policy documents/48-国家信访局党组关于印发《国家信访局机关工作人员请假休假管理办法》的通知(2019)(FBM-CLI-16-332044).doc]

国家信访局党组关于印发《国家信访局机关工作人员请假休假管理办法》的通知(2019)

**发布部门：** [国家信访局](https://www.pkulaw.com/law/chl?Aggs.IssueDepartment=7060353)

**发文字号：**国信党发〔2019〕7号

**发布日期：**2019.03.18

**实施日期：**2019.03.18

**时效性：** [现行有效](https://www.pkulaw.com/law/chl?Aggs.TimelinessDic=01)

**效力级别：** [党内法规制度](https://www.pkulaw.com/law/chl?Aggs.EffectivenessDic=XR12)

**法规类别：** [信访](https://www.pkulaw.com/law/chl?Aggs.Category=00305)

国家信访局党组关于印发《国家信访局机关工作人员请假休假管理办法》的通知
（国信党发〔2019〕7号）

各党总支、直属党支部，各司、室、中心：
　　《国家信访局机关工作人员请假休假管理办法》已经2019年3月15日国家信访局党组第10次会议讨论通过，现印发给你们，请认真贯彻执行。

中共国家信访局党组
2019年3月18日

　　国家信访局机关工作人员请假休假管理办法

　第一章　总　则

　　**第一条**　为进一步规范和加强局机关工作人员请假、休假管理，根据有关规定和我局实际，制定本办法。

　　**第二条**　本办法适用于局机关在编工作人员请假、休假管理。挂职、借调等编外人员的请假、休假事宜，由用人司（室、中心）依据国家有关规定，参照本办法进行管理。

　　**第三条**　司（室、中心）应在确保完成工作任务的前提下，统筹安排，落实好工作人员享有的休假待遇；工作人员因故请假的，要根据工作情况和本人实际需要，加强审批管理，妥善予以安排。离京外出的，须履行相应审批手续。
　　工作人员请休假情况纳入平时考核及年度考核。

　　**第四条**　工作人员请假、休假期间，应遵守党纪国法和机关各项纪律要求。

　第二章　假期期限和待遇

　　**第五条**　年节及纪念日假
　　（一）全体工作人员假
　　元旦、春节、清明节、劳动节、端午节、中秋节、国庆节等国家统一规定的假日和北京市临时规定的假日，全体工作人员按有关通知安排放假。
　　（二）部分工作人员假
　　妇女节，女同志放假半天；
　　青年节，28周岁（含）以下青年放假半天。
　　以上假日如逢星期六、星期日，不补假、不调休。

　　**第六条**　事假
　　工作人员因私事不能正常工作，应当向所在司（室、中心）请假，由所在司（室、中心）根据工作情况和本人实际需要确定假期期限。请事假的，冲抵年休假假期，冲抵年休假期的事假不纳入事假考勤统计。

　　**第七条**　病假
　　（一）工作人员因病不能正常工作，须持医院或局医务室出具的病假证明，申请休病假。病假证明应及时交所在司（室、中心）审核，并在每月考勤汇总时提供给人事部门备案。
　　（二）工作人员病假期间工资待遇按以下标准执行：
　　1. 病假在2个月以内的，发给原工资。
　　2. 病假超过2个月且不满6个月的，从第3个月起按下列标准发给病假期间工资：工作年限不满10年的，基本工资按90%计发；工作年限满10年的，工资全额照发。
　　3. 病假超过6个月的，从第7个月起按下列标准发给病假期间工资：工作年限不满10年的，基本工资按70%计发；工作年限满10年的，基本工资按80%计发。

　　**第八条**　婚、丧假
　　（一）依法办理结婚登记的工作人员，婚假一般为10天。配偶在外地并在外地结婚的，可酌情给予往返路程假。
　　（二）工作人员直系亲属（父母、岳父母、公婆、配偶及子女）去世的，假期一般为3天。去世的亲属在京外的，可酌情给予往返路程假。

　　**第九条**　产假
　　（一）女职工正常分娩假期98天，其中产前可以休假15天。难产的增加产假15天。多胞胎生育的每多生育1个婴儿增加产假15天。按规定生育的，另外享受生育奖励假30天。
　　局机关男职工配偶按规定生育子女的，本人享受陪产假15天。
　　女职工申请休生育奖励假和增加产假，男职工申请休陪产假，应按程序报批，并将医院有关证明和生育登记资料提供给人事部门备案。
　　（二）女职工怀孕不满4个月流产的，享受15天产假；怀孕满4个月以上流产的，享受42天产假。
　　（三）产假结束后，对哺乳未满1周岁婴儿的女职工，每天安排1个小时哺乳时间；女职工生育多胞胎的，每多哺乳1个婴儿每天增加1小时哺乳时间。
　　（四）女职工休产假期间工资待遇不变。产假期满，因身体原因仍不能工作的，其超过产假期间的待遇，按病假有关规定办理。
　　以上假期均含公休假日和法定节日。

　　**第十条**　探亲假
　　（一）工作满1年的工作人员，与配偶分居两地，又不能在公休假日团聚的，可享受探望配偶假，申请时须提供对方单位证明材料；与父母（不包括岳父母和公婆）双方分居两地，又不能在公休假日团聚的，可享受探望父母假。
　　（二）探亲假期
　　1. 已婚工作人员探望配偶，其中一方每年享受探亲假1次，假期30天。
　　2. 未婚工作人员探望父母，每年享受探亲假1次，假期20天。因工作需要当年未探亲或工作人员自愿两年探亲1次的，假期45天。
　　3. 已婚工作人员探望父母，每4年享受探亲假1次，假期20天。
　　（三）工作人员丧偶或离异后未再婚的，在丧偶或离婚满1年后可按未婚工作人员探望父母的规定享受探亲假。其中，上半年满1年的，可在满1年以后的当年享受探亲假待遇；下半年满1年的，从下年度起享受探亲假待遇。
　　以上假期均含公休假日和法定节日。

　　**第十一条**　带薪年休假
　　（一）连续工作时间满1年以上的工作人员，享受带薪年休假。
　　（二）工作年限满1年、满10年、满20年后，从下月起享受相应的年休假天数：
　　1. 工作年限满1年不满10年的，年休假5天；
　　2. 工作年限满10年不满20年的，年休假10天；
　　3. 工作年限满20年的，年休假15天。
　　公休假日、法定节日和探亲假、婚丧假、产假的假期，不计入年休假的假期。
　　（三）工作人员有下列情形之一的，不享受当年的年休假：
　　1. 累计工作满1年不满10年的工作人员，请病假累计2个月以上的；
　　2. 累计工作满10年不满20年的工作人员，请病假累计3个月以上的；
　　3. 累计工作满20年的工作人员，请病假累计4个月以上的。
　　（四）工作人员有下列情形之一的，不享受下一年的年休假：
　　1. 当年已享受年休假，年内又出现本条第三款情形之一的；
　　2. 当年的年休假期已休满或者已冲抵完，请事假天数又超过本人可享受年休假天数的。
　　（五）年休假原则上在每年8月份集中安排、一次性休完，确因工作需要最多分两段休完，但不能跨年度安排。确因工作需要不能安排年休假的，对未休假天数，参照加班费有关标准给予补助。

　第三章　审批和备案

　　**第十二条**　工作人员请假、休假，应按照职务层次填写相应的《国家信访局机关工作人员请假、休假、离京审批表》，按以下程序进行审批。
　　（一）局党组成员、副局长请假、休假，由局长批准；
　　（二）司局级干部请假、休假，由所在司（室、中心）与人事部门共同审核，报经分管局领导同意后，由局长批准；
　　（三）正处级干部请假、休假，经处长、分管司领导、司长（主任）同意后，由分管局领导批准；
　　（四）副处级及以下工作人员请假、休假，经处长、分管司领导同意后，由司长（主任）批准。

　　**第十三条**　工作人员请假、休假、离京应当至少提前3个工作日申请，待批准后方可离开工作岗位。因特殊情况无法提前申请的，应注明原因；本人不在机关的，应及时委托他人代办手续。未经批准离开工作岗位的，按旷工处理。

　　**第十四条**　工作人员请假、休假审批完毕后，应及时报人事部门备案；假期结束返回局机关后，应及时销假。

　　**第十五条**　存在下列情形之一的，工作人员所在司（室、中心）应在情况出现10个工作日内，将请假原因、期限报人事部门备案，人事部门按国家有关规定提出相关意见，报局领导审批后按有关规定执行：
　　（一）工作人员请病假连续超过2个月和6个月的；
　　（二）工作人员1年内请事假、病假累计超过6个月的；
　　（三）工作人员因私事出国（境）超过规定假期期限的。

　　**第十六条**　各司（室、中心）应于每月5日前，将本部门工作人员上月请假、休假情况报人事部门备案；每年1月份，将本部门工作人员上年度请假、休假情况报人事部门备案。

　第四章　管理责任

　　**第十七条**　局党组负总责，分管人事的副局长是直接责任人，负责全局工作人员的请假、休假管理工作。

　　**第十八条**　各司（室、中心）主要负责同志为本部门请休假管理第一责任人，要认真履行主体责任和管理职责。安排专人负责请假、休假情况汇总，并在平时考核和年终考核时通报点评。

　　**第十九条**　人事部门是请假、休假的主管部门，负责对各司（室、中心）报备的人员请假、休假情况进行审核，检查、督促各司（室、中心）认真贯彻执行本规定。

　第五章　附　则

　　**第二十条**　本办法所规定的假期期限和待遇，如国家有新规定，按新规定执行。

　　**第二十一条**　本办法由人事部门负责解释。

　　**第二十二条**　本办法自印发之日起施行。2016年12月15日印发的《国家信访局机关工作人员请假休假管理办法》同时废止。

© 北大法宝：（[www.pkulaw.com](https://www.pkulaw.com/)) 专业提供法律信息、法学知识和法律软件领域各类解决方案。北大法宝为您提供丰富的参考资料，正式引用法规条文时请与标准文本核对。
欢迎查看所有[产品和服务](https://www.pkulaw.com/helps/2.html)。法宝快讯：[如何快速找到您需要的检索结果？](https://www.pkulaw.com/helps/70.html)[法宝](https://www.pkulaw.com/helps/69.html)V6有何新特色？

扫描二维码阅读原文

**原文链接：**<https://www.pkulaw.com/chl/e70997300fb1ce8fbdfb.html>
